# Effect of Epigallocatechin-3-gallate on Stress-Induced Depression in a Mouse Model: Role of Interleukin-1β and Brain-Derived Neurotrophic Factor

**DOI:** 10.1007/s11064-022-03707-9

**Published:** 2022-08-08

**Authors:** Nabila E. Abdelmeguid, Tasneem M. Hammad, Ashraf M. Abdel-Moneim, Sherine Abdel Salam

**Affiliations:** 1 Department of Zoology, Faculty of Science, Alexandria University, Alexandria, 21511 Egypt; 2 Department of Medical Laboratory Technology, Faculty of Applied Health Sciences Technology, Pharos University, Alexandria, Egypt

**Keywords:** Epigallocatechin-3-gallate (EGCG), Chronic unpredictable mild stress (CUMS), Antidepressant, Interleukin-1β (IL-1β), Brain-derived neurotrophic factor (BDNF), Hippocampal pathology

## Abstract

Epigallocatechin 3-gallate (EGCG) is a natural polyphenolic antioxidant in green tea leaves with well-known health-promoting properties. However, the influence of EGCG on a chronic animal model of depression remains to be fully investigated, and the details of the molecular and cellular changes are still unclear. Therefore, the present study aimed to investigate the antidepressant effect of EGCG in mice subjected to chronic unpredictable mild stress (CUMS). After eight consecutive weeks of CUMS, the mice were treated with EGCG (200 mg/kg b.w.) by oral gavage for two weeks. A forced swimming test (FST) was used to assess depressive symptoms. EGCG administration significantly alleviated CUMS-induced depression-like behavior in mice. EGCG also effectively decreased serum interleukin-1β (IL-1β) and increased the mRNA expression levels of brain-derived neurotrophic factor (BDNF) in the hippocampal CA3 region of CUMS mice. Furthermore, electron microscopic examination of CA3 neurons in CUMS mice showed morphological features of apoptosis, loss or disruption of the myelin sheath, and degenerating synapses. These neuronal injuries were diminished with the administration of EGCG. The treatment effect of EGCG in CUMS-induced behavioral alterations was comparable with that of clomipramine hydrochloride (Anafranil), a tricyclic antidepressant drug. In conclusion, our study demonstrates that the antidepressive action of EGCG involves downregulation of serum IL-1β, upregulation of BDNF mRNA in the hippocampus, and reduction of CA3 neuronal lesions.

## Introduction

Major depressive disorder (MDD) is considered to be a major contributing factor to health-related disability and a risk factor for suicide [1]. Ranging from mild to severe, MDD affects approximately 6% of new patients every year, and even after multiple treatment attempts nearly 30% of patients do not remit MDD [2]. A biomarker-based approach is presently used to support clinical diagnostic criteria and identify neurobiological mechanisms that could mediate different MDD endophenotypes [3]. There is substantial evidence that inflammatory disturbances may be intimately involved in psychiatric symptoms [4]. Recent pharmacological experiments and clinical observations have shown that MDD patients display higher circulating levels of proinflammatory cytokines [5]. Much of the interest in inflammation and depression has been focused on cytokines that mediate the innate immune response, such as interleukin-1beta (IL-1β). In fact, increased levels of IL-1β impair hippocampal neurogenesis either by dysregulating the HPA axis or by inhibiting proliferation via cell cycle arrest [6]. This indicates that under conditions of immune activation, the production of IL-1β significantly contributes to the induction of MDD [7]. Therefore, IL-1β can be used as a therapeutic target for innovative MDD treatments [8]. In addition, neuroimaging and postmortem studies on patients diagnosed with depression have revealed changes in the anatomy and functionality in various brain regions, including the amygdala, thalamus, hippocampus, and prefrontal cortex (PFC) [9]. MDD-induced alterations in these brain regions could be a result, in part, of the reduced expression levels of brain-derived neurotrophic factor (BDNF) [10]. BDNF is a brain protein that contributes to the survival, development, and maintenance of neurons and is involved in several learning and memory functions [11]. Indeed, BDNF is considered to be one of the central players in the pathogenesis of MDD [12].

Most of the current medications used to treat MDD belong to a class of drugs called tricyclic antidepressants, which act by inhibiting transporters for serotonin and/or noradrenaline [13]. Among these, Anafranil (the commercial name of clomipramine hydrochloride) is on the list of the essential medicines approved by the U.S. Food and Drug Administration [14]. Clomipramine inhibits B-lymphocyte activity, ameliorates clinical signs of experimental autoimmune encephalomyelitis, reduces inflammation and microglial activation, and preserves axonal integrity [15]. However, clomipramine has many side effects, including nausea, weight increase, sexual dysfunction, sedation, hypotension, dry mouth, sweating, obstipation, blurred vision, and micturition [16]. Therefore, safe therapies for depression are still needed. In this regard, the use of phytomedicine may be an alternative option to develop new active herbal drugs for the treatment of depression with higher potency and lower toxicity [17]. There has been a worldwide increase in the use of complementary and alternative medicine for most physical and mental health problems, and a few natural products with potential psychiatric applications have been studied [18, 19]. Over the last decade, epigallocatechin-3-gallate (EGCG), the most abundant polyphenol derived from green tea leaves, has received a great deal of interest as a possible therapeutic agent for the prevention of neurodegenerative, inflammatory, and cancer diseases [20, 21]. The literature regarding the anti-stress effects of EGCG suggest that EGCG may restore HPA activity via ERK upregulation, decrease corticosterone/CRH/ACTH, reduce neuron overexcitation, and improve GABA transmission via activation of the SIRT1/PGC-1α pathway [22]. In addition, Li et al. [23] indicated that EGCG could ameliorate depressive syndromes in rats by enhancing serotonin (5-HT) levels and neuroprotection in the hippocampus. EGCG is a powerful candidate for suppression of the immune/inflammatory response and apoptosis in multiple cellular and animal models [24] and thus can contribute to the mitigation of depression [25, 26].

In animal research, chronic unpredictable mild stress (CUMS) is a classic, established model that has been widely applied for researching depressive disorders and antidepressants [27, 28]. Importantly, the CUMS paradigm closely mimics a number of behavioral and neuroendocrine alterations in depressive patients [29]. Therefore, in the present study, CUMS model albino mice were used to investigate the antidepressive effects of EGCG. Our results provide new insights into some of the bases for EGCG action in depression, which we hypothesize may involve modulation of IL-1β peripheral blood levels, BDNF expression in the hippocampus and neuronal ultrastructural damage in CUMS disorder.

## Materials and Methods

### Animals

Twenty-four male Swiss albino mice weighing 30 − 40 g were purchased from Pharos University, Alexandria, Egypt. Mice were kept under standardized conditions of a 12:12 light:dark cycle at 25 ± 3 °C and 35%–60% relative humidity. Pelleted mouse feed and water were provided ad libitum. All procedures and animal handling methods throughout this work were approved by the animal ethics committee of Alexandria University (ALEXU-IACUC Ref. No. AU-04–18-12–26-MSc-1 dated 26 Dec. 2018), in accordance with ICLAS guidelines for the ethical care of experimental animals.

### Chemicals and Reagents

EGCG powder (purity 99.91%) was purchased from MedChemExpress, Monmouth Junction, NJ, USA (Cat. No.: HY-13653). Anafranil capsules were acquired from Novartis Pharma Company, Cairo. Other reagents were of the highest analytical grade.

### Stress Model

Mice of the four test groups (excluding the controls) were housed in individual cages and exposed to the CUMS protocol for 8 weeks [30]. The protocol included the daily exposure to a single random stressor. Stress procedures included (1) 2 h of physical restraint in a 50 ml plastic tube (Falcon) with openings in both sides for breathing, (2) 10 min exposure to hot air steam from a hairdryer on a low setting, (3) 3–4 h with damp bedding, (4) 6 h overnight illumination, (5) 4 h tilting the mouse's cage at a 45° from the horizontal, (6) 10 min warm water forced swimming, (7) 5 min cold water (4 °C) swimming, after which the mice were toweled dry, and (8) 24 h light/dark alterations. All stressors were randomly scheduled over a one-week period and repeated throughout the eight-week experiment (Table 1) to ensure the unpredictability of the stressors.

**Table 1:** The CUMS protocol-induced depression mouse model

Day	Stressor	Duration
Saturday	Physical restraint	2 h
Sunday	Hot air steam	10 min
Monday	Damp bedding	3–4 h
Tuesday	Overnight illumination	6 h
Wednesday	Warm water swimming	10 min
	or cold water (4 °C) swimming	5 min
Thursday	Tlting cage	3–4 h
Friday	Light/dark alterations	24 h

### Experimental Design

After two weeks of acclimatization, the mice were randomly assigned to four groups (6 animals each) as follows:

- Control group: Animals did not receive any treatment.
- CUMS mouse model: Mice were subjected to CUMS for 8 weeks.
- CUMS + EGCG group: CUMS mice were orally administered EGCG at a dose of 200 mg/kg b.w. dissolved in 0.2 ml normal saline for two weeks (D63—D77).
- CUMS + Anafranil group: CUMS mice were orally administered Anafranil (a reference drug) at a dose level of 20 mg/kg b.w. dissolved in 0.2 ml normal saline for two weeks (D63—D77).

**Fig. 1:**
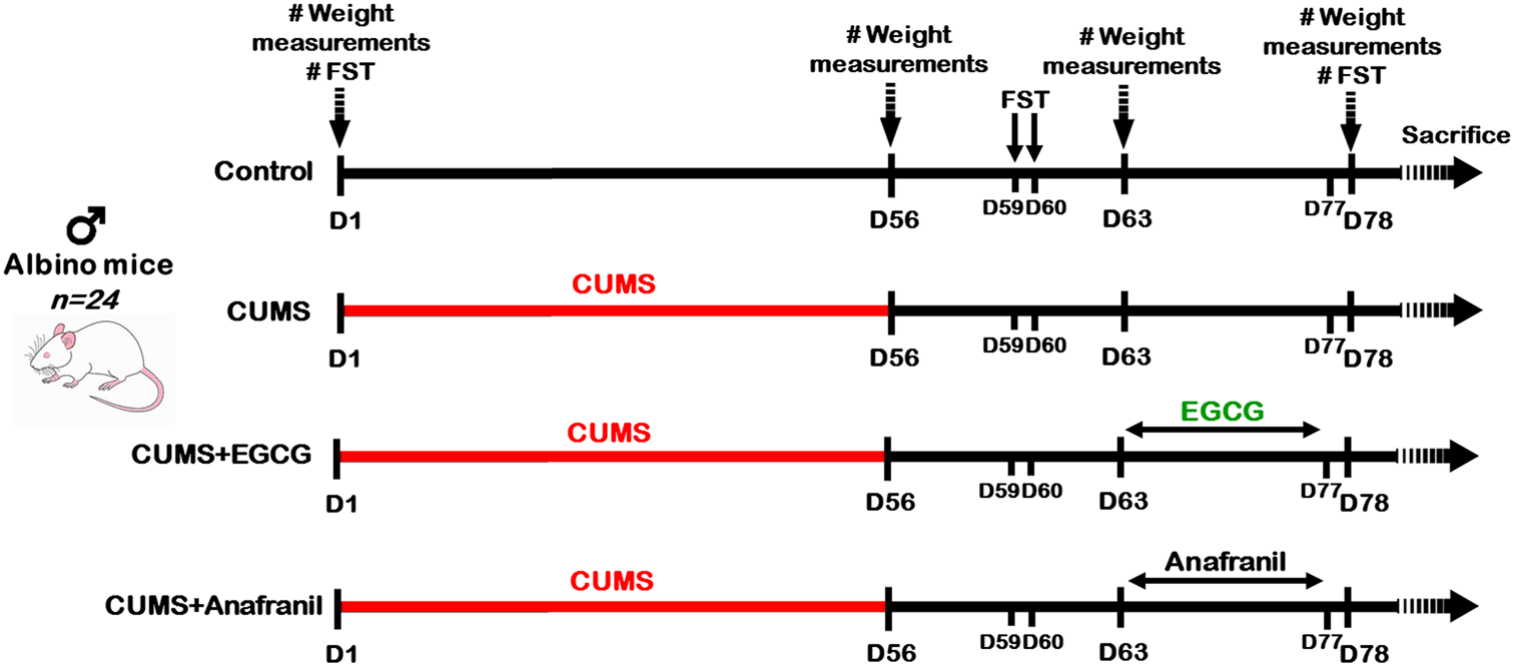
Timeline of the experimental design. The CUMS protocol consisted of the application of a variety of stressor stimuli for 8 weeks. EGCG (200 mg/kg b.w.) and Anafranil (20 mg/kg b.w) were orally administered for 2 weeks after the CUMS procedure

The doses of EGCG and Anafranil were chosen based on previously published studies [31–33]. Animal weights were measured at the beginning of the experiment (D1), at the end of CUMS induction (D56), on the first day of therapy (D63), and at the end of therapy (D78). Behavioral tests (i.e., forced swimming test) were conducted on D1, after CUMS induction (D59 and D60), and on D78. Then, all mice were sacrificed on D79. Each experimental animal was sublethally anesthetized by intraperitoneal injection with a mixture of ketamine (100 mg/kg b.w.) and xylazine (5 mg/kg b.w.). For transmission electron microscopy (TEM), three mice from each group were perfused through the left ventricle of the heart with 4% paraformaldehyde (PFA) solution in 0.01 M phosphate buffer (PB; 100 ml for each mouse) [34]. After perfusion, the entire brain of each animal was removed, and the hippocampus was dissected and placed into vials containing a mixture of formalin-glutaraldehyde fixative (_4_F_1_G) fixative. Fresh samples were obtained from the other three animals for ELISA and qRT–PCR measurements. For ELISA, blood samples were collected in anticoagulant-free tubes, incubated at room temperature for 20 min, and then centrifuged at 1000–2000×g for 10 min at 4 °C. The supernatant (serum) was immediately aliquoted and stored at − 20 °C before the IL-1β assay. For qRT–PCR, the hippocampus was isolated, placed in Eppendorf tubes, and subsequently stored at − 80 °C until further analysis of BDNF gene expression. The whole experimental protocol is shown in Fig. 1.

### Forced Swimming Test (FST)

The FST test involved assessing passive (immobility) behavior in mice as described previously [35, 36]. In brief, six animals from each group were separated and placed in the testing room for at least one hour to acclimate them to the new environment. The mice were then forced to swim for 6 min in an inescapable transparent cylindrical tank (30 cm height × 20 cm diameters) filled with 15–20 cm of water at a temperature of 23–25 °C. The first 2 min are considered the habitation period. The total duration of immobility was calculated in seconds during the subsequent 4 min. A mouse was judged to be immobile whenever it stopped struggling and remained floating passively in the water with its head just above the water level. The mice were then dried and removed to their cages.

### Assessment of Serum IL-1β

Serum IL-1β was determined by a mouse IL-1β simple step ELISA Kit (Cat. No. ab197742, Abcam, United Kingdom). The kit has a detection limit of 1 pg/ml. Fifty microliters of standard solution or the samples were added to appropriate wells in the ELISA plate. Subsequently, 50 µl of the antibody cocktail was added to each well, and the samples were incubated for one hour at room temperature. After washing three times with 350 ​μl of 1X wash buffer PT, the plate was inverted and blotted against clean paper towels to remove excess liquid, and 100 µl of TMB development solution was added to each well and incubated for 10 min in the dark on a plate shaker set to 400 rpm. Then, 100 µl of stop solution was added, and the plate was shaken on a plate shaker for 1 min to mix. The optical density was recorded at 450 nm, as an endpoint reading. The standard curve for the target protein was created by plotting the mean absorbance (nm; y-axis) against the protein concentration (pg/ml; x-axis) and used to determine the concentration of target protein in each sample.

### Assessment of BDNF Expression in the Hippocampus

qRT–PCR was used to determine the expression level of BDNF mRNA in the hippocampus. Approximately 50–100 mg of hippocampal tissue was used for total RNA isolation. Tissue homogenization and RNA isolation were performed using the easy-spin total RNA extraction kit (Cat No. 17551, Intron Biotechnology, Korea) according to the manufacturer's instructions. Isolated RNA was then transcribed by reverse transcriptase into complementary DNA (cDNA) and used as the qRT–PCR reaction template using a cDNA reverse transcription kit (Cat No. 4374966, Thermo Fisher, USA) as instructed by the manufacturer. For the amplification step, primer sets and probes from the TaqMan gene expression assay (Cat No. 4331182, Thermo Fisher, USA) for BDNF (Mm04230607_s1), GAPDH (Mm99999915_g1), and TaqMan master mix (Cat No. 203446, Thermo Fisher, USA) were used. Mouse GAPDH was used as a housekeeping gene. The mRNA expression level for the four groups from qRT–PCR experiments was calculated using the 2^−ΔΔC*T*^ method to measure relative changes in mRNA expression [37].

### TEM Ultrastructural Examination

_4_F_1_G-fixed samples of hippocampal CA3 were postfixed in 1% osmium tetroxide (OsO_4_), dehydrated in ethanol, infiltrated in propylene oxide and embedded in an Epon-Araldite mixture. Ultrathin Sects. (70 nm thick) were cut using a glass knife with an LKB ultratome (LKB Bromma, Austria), double-stained with uranyl acetate and lead citrate, picked on 200 mesh naked copper grids, and examined under a Jeol 100CX TEM operating at 80 kV (Jeol Ltd., Japan).

### Statistical Analysis

All analyses were performed using IBM SPSS statistics version 20.0 (IBM Corp., Armonk, NY), and the data are expressed as the means ± standard errors (SEs). Before statistical analysis, all variables were verified for normality using the Kolmogorov–Smirnov (K-S) test. If the K-S test indicated insignificant deviation, the obtained data were analyzed by one-way or repeated-measures ANOVA followed by a post hoc test for multiple comparisons (Tukey). Variables subjected to ANOVA include weight measurements, ELISA, and qRT–PCR results. In the case of significant deviation in the K-S test, analysis of the time effect was performed using Friedman’s test (a nonparametric form of repeated-measures ANOVA). In addition, the Kruskal–Wallis test was used to analyze the between-group treatment effect, and Dunn's multiple comparisons test was conducted as a post hoc test to determine the specific pairs of groups that were significantly different. Nonparametric analyses were performed on FST results*.* A *p* value < 0.05 was considered statistically significant.

## Results

### EGCG Ameliorates the Negative Effect of CUMS on Body Weight

The data in Table 2 depict the body weights of mice under different treatments. Repeated measures ANOVA on the mouse weight over the time points yielded a significant main effect of time (F = 56.495, *p* < 0.001) and time x treatment interaction (F = 55.419, *p* < 0.001). Mice subjected to CUMS showed signs of appetite loss and a gradual decrease in body weight throughout the induction period (D1-D56). On D78, the body weight of mice significantly decreased in the CUMS mice when compared with the control (− 24.89%, one-way ANOVA, F = 25.667, *p* < 0.001). Oral administration of EGCG (200 mg/kg b.w.) for two weeks insignificantly reversed this decline (+ 4.41%) when compared with CUMS alone. The reference drug Anafranil (20 mg/kg b.w.) significantly blocked the loss of body weight caused by CUMS (+ 24.30%). Additionally, post hoc analysis indicated a slight but significant increase in the weights of CUMS + EGCG (+ 9.87%) and CUMS + Anafranil-treated mice (+ 8.16%) starting from drug administration (D63) until D78.

**Table 2:** Effect of EGCG and Anafranil on body weights in CUMS-exposed mice

Groups	Day 1	Day 56	Day 63	Day 78
Control	33.0 ± 0.63	34.5 ± 0.85	35.83 ± 0.65	40.17 ± 1.25
CUMS	36.5 ± 0.92*	34.33 ± 0.95	32.83 ± 0.98*	30.17 ± 1.08*
CUMS + EGCG	35.83 ± 0.70	31.67 ± 0.67	28.67 ± 0.49*#	31.50 ± 0.43*
CUMS + Anafranil	40.0 ± 0.68*#	36.33 ± 0.67	34.67 ± 0.61	37.50 ± 0.81#

Values are expressed as mean ± SE for six mice in each group

*p < 0.05 compared with control at the same time point

^#^*p* < 0.05 compared with CUMS at the same time point

### EGCG Restores CUMS-induced Depression-like Behavior

**Fig. 2:**
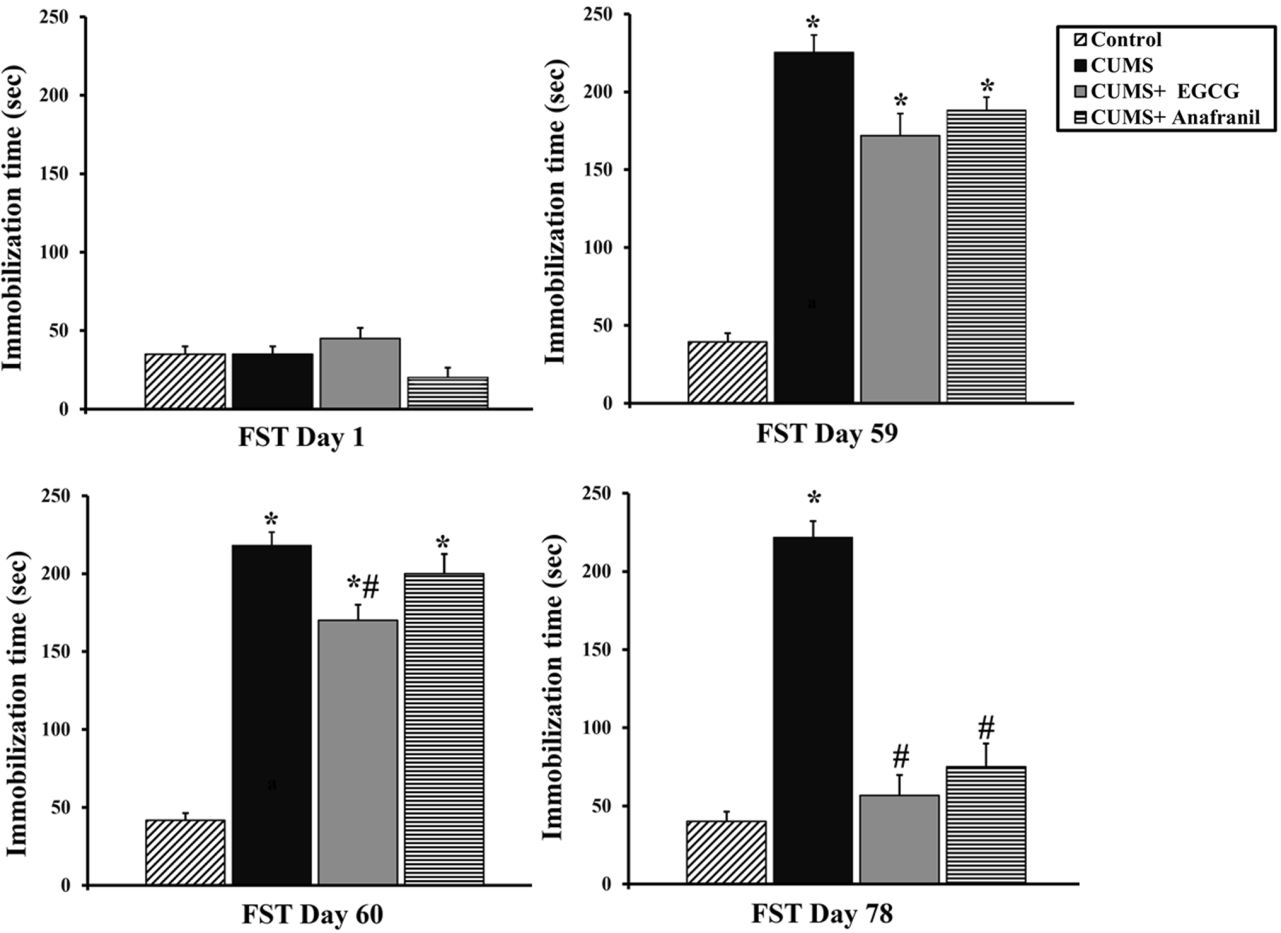
Effect of EGCG and Anafranil on the immobility time caused by CUMS during the FST. The bars represent the means ± SEs (*n* = 6). *p < 0.05 compared with control at the same time point; ^#^ *p*< 0.05 compared with CUMS at the same time point

Next, the FST was performed to assess depression-like behavior in all experimental groups (Fig. 2). Exposure to CUMS for 8 weeks clearly resulted in depression-like behavior in the FST (Friedman test for time-dependent effect, CUMS: Q = 13.118, *p* = 0.004; CUMS + EGCG: Q = 15.421, *p* = 0.001; CUMS + Anafranil: Q = 16.966, *p* = 0.001). On D78, the immobility time was severely and significantly increased (+ 454.25%) in the CUMS mice when compared with the nonstressed control mice (Kruskal–Wallis test, Q = 15.974, *p* = 0.001). However, the CUMS-induced increase in immobility time was significantly lower in CUMS mice treated with EGCG (− 74.44%) and Anafranil (− 66.17%) than in the CUMS mouse model, and the values were reversed to the same levels as those of the control mice. Thus, these findings indicate a potential antidepressant action of EGCG in this CUMS model, and that its effect was similar to that of the reference drug (i.e., Anafranil).

### EGCG Decreases Serum IL-1β in CUMS Mice

**Fig. 3:**
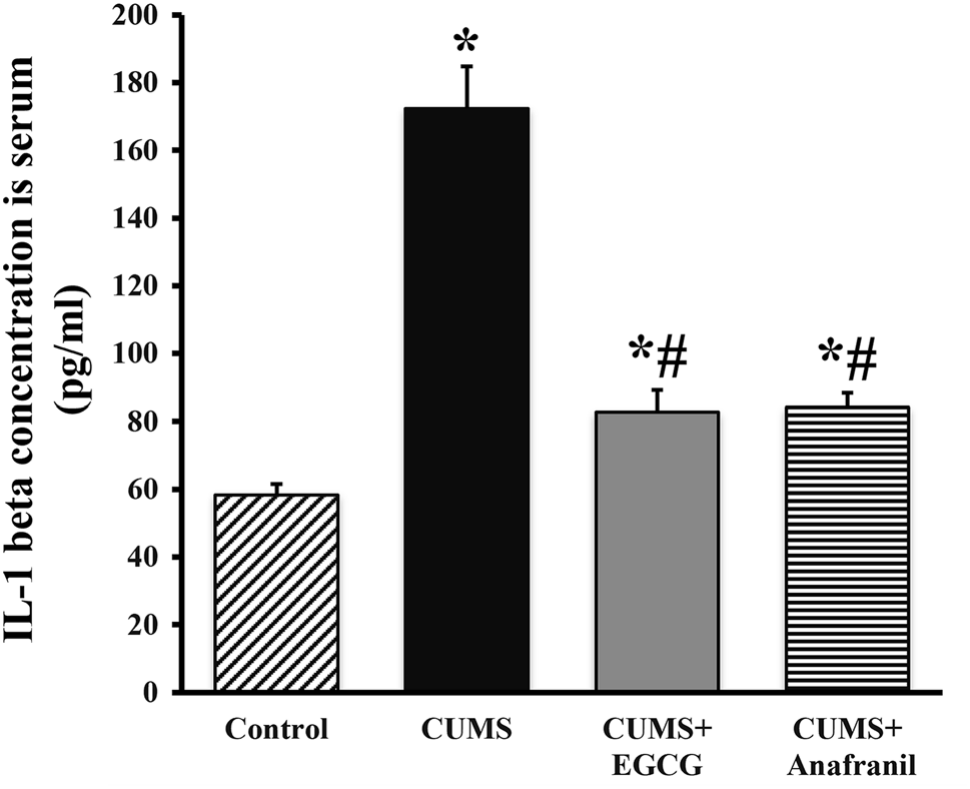
ELISA results showing the effect of EGCG and Anafranil on the production of serum IL-1β by the immune system in CUMS-exposed mice. The bars represent the means ± SEs (*n* = 3). *p < 0.05 compared with control; ^#^*p* < 0.05 compared with CUMS

Neuroinflammation is considered to be an important underlying process in the pathophysiology of MDD [38]. One-way ANOVA showed a significant difference in the IL-1β values among the experimental groups (F = 37.810, *p* < 0.001). As shown in Fig. 3, ELISA analysis showed that the expression level of the proinflammatory factor IL-1β in mice exposed to CUMS was significantly increased (+ 195.44%) when compared with the controls. This increase was significantly inhibited by treatment with EGCG (− 52.03%) and Anafranil (− 51.26%) and almost returned to normalcy. These results show that EGCG considerably decreased the inflammatory response after CUMS challenge.

### EGCG Increases Hippocampal BDNF mRNA in CUMS Mice

**Fig. 4:**
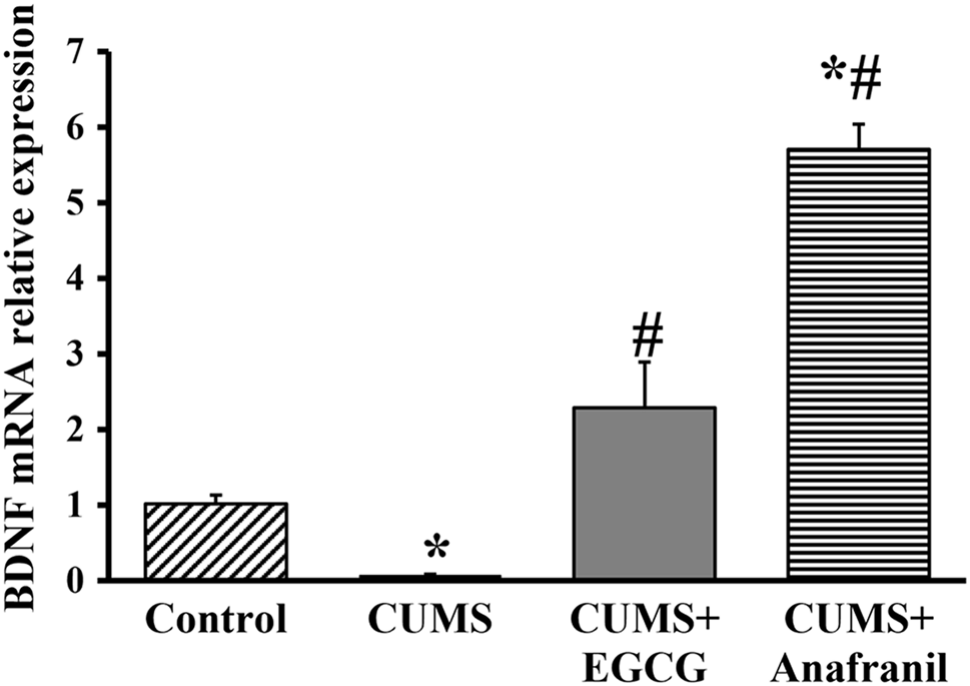
qRT–PCR results showing the effect of EGCG and Anafranil on the hippocampal BDNF level in CUMS-exposed mice. The bars represent the means ± SEs (*n* = 3). *p < 0.05 compared with control; ^#^*p* < 0.05 compared with CUMS

Chronic stress and depression are known to impair BDNF synthesis in the hippocampus and cerebral cortex [39, 40]. With this in mind, we examined the possible involvement of BDNF in the antidepressant effect induced by EGCG in CUMS mice. Using qRT–PCR, BDNF mRNA expression in the hippocampus was recorded and quantified in all experimental groups (Fig. 4). The one-way ANOVA for BDNF showed a significant effect of treatments (F = 86.231, *p* < 0.001). As expected, we found that the BDNF mRNA level was 93.14% lower in CUMS-exposed mice than in control mice. Notably, CUMS-exposed mice treated with EGCG showed a significantly increased expression level of BDNF (approx. by 30-fold) when compared with the CUMS group. The efficacy of Anafranil in rescuing BDNF levels was superior to that of EGCG. CUMS mice treated with Anafranil expressed a higher BDNF level (approx. by 80-fold) in the hippocampus when compared with the CUMS-exposed mice.

### EGCG Promotes Hippocampal CA3 Neuronal Recovery in CUMS Mice

**Fig. 5:**
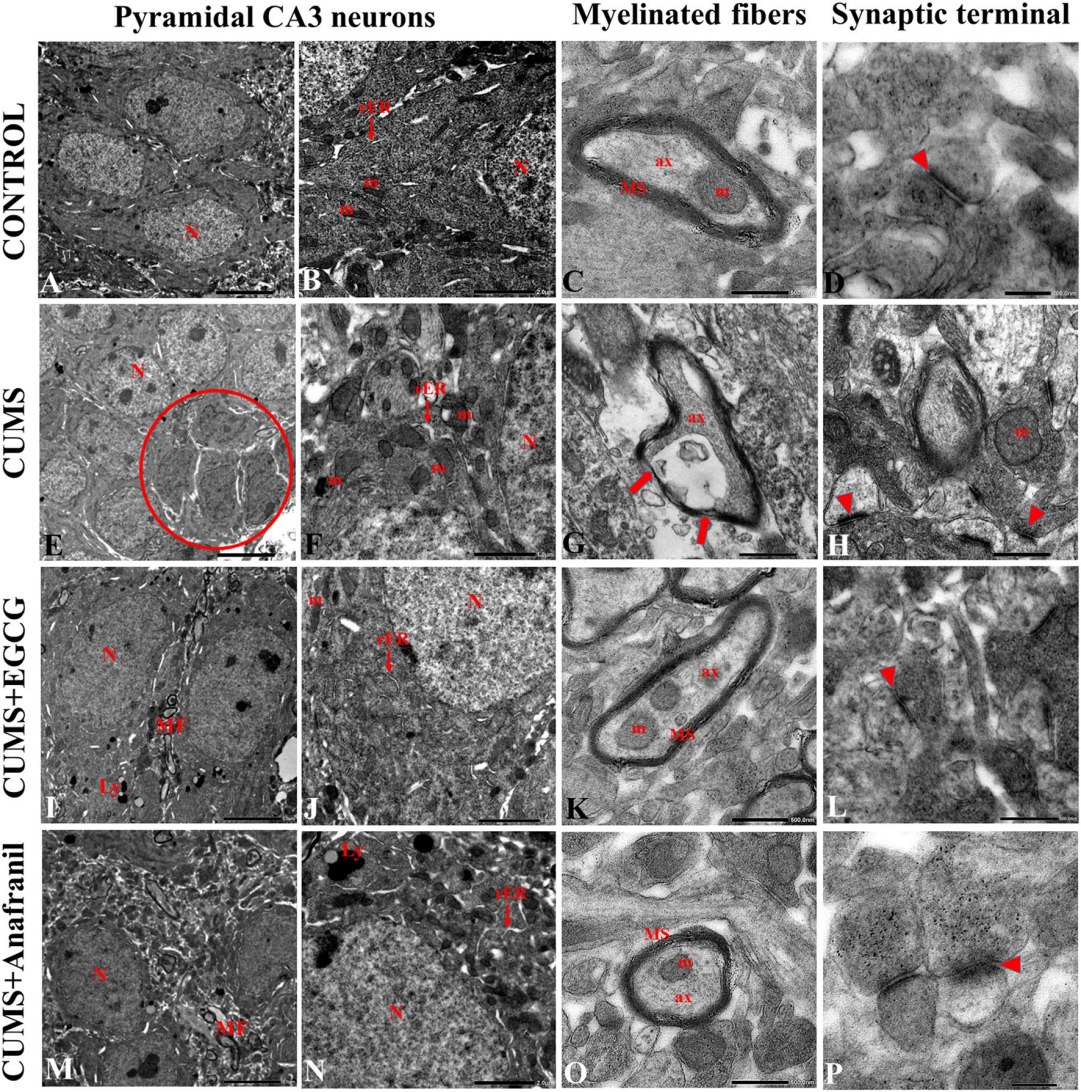
Representative TEM micrographs illustrating the impact of EGCG on hippocampal CA3 neuronal ultrastructure in CUMS-exposed mice. In the control group, the hippocampal CA3 neurons exhibit a normal cell body, with intact organelles and well-formed myelin sheaths and synaptic elements. In the CUMS group, neuronal degeneration changes include cell shrinkage, nuclear fragmentation, and condensation (red circle). The mitochondria and rER have abnormal morphology, and myelinated nerve fibers are deformed. In addition, hippocampal CA3 synapses depict a decrease in the number of synaptic vesicles and degenerated mitochondria with destroyed cristae, and the synaptic cleft is unclear. However, after EGCG treatment, these signs of degeneration are comparatively lessened. An ameliorative effect is also observed following the application of the reference drug (Anafranil) in CUMS mice. Arrowheads indicate synapses, arrows show loss of myelin lamellae, ax: axoplasm, Ly: lysosomal bodies, m: mitochondria, MF: myelinated fiber, MS: myelin sheath, N: nucleus, rER: rough endoplasmic reticulum. Scale bar: 5 µm (**A**, **E**, **I**, **M**), 2 µm (**B**, **J**, **N**), 1 µm (**F**), 500 nm (**C**, **G**, **H**, **K**, **L**, **O**), 200 nm (**D**, **P**)

Hippocampal damage is a direct reflection of neurodegeneration [41]. In our study, hippocampal (pyramidal) neurons demonstrated a normal ultrastructure image with no signs of apoptosis. In the nucleus, there was finely dispersed chromatin and a regular bilaminar nuclear envelope (Fig. 5A). The cytoplasm was rich in well-arranged organelles, including elongated/spherical mitochondria, rough endoplasmic reticulum (rER), polyribosomes, and Golgi complexes (Fig. 5B). Normal myelinated fibers in the control group were surrounded by regular and tightly wrapped myelin sheaths around axoplasm elements (Fig. 5C). As seen in Fig. 5D, the hippocampus of the control group of mice showed normal synaptic contacts and abundant synaptic vesicles. However, in the CUMS group, a large number of pyramidal neurons showed the structural hallmarks of apoptosis, featuring cell shrinkage, scattered areas of condensed nuclear chromatin, and deformed nuclear envelopes (Fig. 5E). The cytoplasm contained swollen (edematous) mitochondria and dilated rER cisternae with many ribosomes felt off (Fig. 5F). Mice exposed to CUMS exhibited several myelin defects, such as loss of lamellar structure (i.e., demyelination) and adaxonal splitting of myelin lamellae (Fig. 5G). Many axons appeared with irregular outlines and revealed extensive vacuolation. CUMS induced swollen synapses, scarcity in synaptic vesicles, and widened/blurred synaptic clefts (Fig. 5H). Examination of ultrathin sections obtained from the CUMS + EGCG group revealed that most neurons had euchromatic nuclei (Fig. 5I), and the integrity of mitochondria and rER cisternae and their alterations were preserved to near normal state (Fig. 5J). Myelin sheath injury was less evident in CUMS mice that received EGCG, and the myelinated fibers mostly showed a regular arrangement of myelin (Fig. 5K). Furthermore, EGCG ameliorated some of the synaptic abnormalities identified in the CUMS group (Fig. 5L). Likewise, the characteristics of neuronal degeneration induced by CUMS were reduced to some extent after treatment with Anafranil (Fig. 5M). In this group, however, rER alterations were still found, as indicated by dilatation and degranulation (Fig. 5N). In some neurons, CUMS-induced injuries of myelin sheaths were maintained by Anafranil (Fig. 5O). The synaptic structure showed relatively more synaptic vesicles and a distinct cleft in the CUMS + Anafranil group (Fig. 5P) compared to the CUMS-alone exposure group.

## Discussion

To date, MDD is considered to be the second highest contributor to chronic disease burden among all chronic medical cases [42]. Not only is it linked to an increased chance of developing many diseases, such as diabetes mellitus, heart disease, and stroke, but also patients with MDD are 20 times more likely to commit suicide than the healthy population [43]. Recent investigations have used CUMS as a reliable in vivo model to assess MDD and its major symptoms, such as anhedonia and depressive behaviors [44–46]. Several studies have illustrated the negative effect of the CUMS protocol on the body weight and appetite of experimental animals, and this weight loss has been reported to be reversed using antidepressant drugs [47–49]. Data obtained in the current study showed that periodic measurements of the body weight of the animals were in accordance with these previous findings. However, treatment with EGCG did not significantly modify the weight loss in comparison with the CUMS animal model (on D78). Nevertheless, the body weight of CUMS + EGCG mice increased significantly from D63 to D78. These results are consistent with a recent study by Zhu et al. [50] showing that body weight increased in a gastric carcinoma rat model after oral treatment with EGCG at a dosage of 50 mg/kg. However, the effects of EGCG on reduced adiposity and improved metabolic profiles are still controversial [51]. Data from another study showed that EGCG supplementation for 20 weeks may be effective in reducing obesity and adipose tissue mass in mice [52]. The proposed mechanisms of EGCG on body weight involve its negative action on intestinal lipid and protein absorption, thus increasing calorie loss besides its enhancement of lipid metabolism via AMP-activated protein kinase (AMPK) in white fat [52, 53].

Here, to assess depression-like behaviors in mice, the FST was used to confirm the establishment of the CUMS model [54]. A strong body of evidence has supported the idea that chronic stress induced for a short period of time results in an increase in the immobility time of experimental animals [55, 56]. This finding is consistent with our work. Despite certain limitations, FST is an effective tool for measuring the pharmacological effects of antidepressants or changes in stress-evoked behavior in preclinical experiments, and it has been extensively used in the field of behavioral neuroscience [57, 58]. However, even if the FST has predictive validity to evaluate responses to antidepressants, one should always consider that the FST is not a full spectrum analog of human depression [59, 60], and it assesses only one aspect of depressed mood, i.e., despair-like behavior. According to previous studies, this behavior might reflect a remarkable adaptation that allows animals to survive in a undesirable situation without unnecessary energy expenditure [61]. However, these concerns should not preclude the usefulness of FST as a suitable screening test in the preliminary search for novel compounds with potential antidepressant activity [62]. Our data indicated that EGCG significantly decreased the immobility time during the FST, reversing the despair behavior caused by CUMS. EGCG seemed to have a beneficial effect on mood. In an earlier study using CUMS rats, intragastric treatment with EGCG (50 mg/kg) was also able to improve anhedonic behavior, another core symptom of depression [25].

Since inflammation has been proposed to promote the development of depressive mood episodes [63], we assessed the inflammatory cytokine IL-1β. The results showed that a CUMS protocol application for 8 weeks (56 days) resulted in a significant increase in mouse serum IL-1β concentration, which is in line with previous studies [7, 64, 65]. Furthermore, the proinflammatory cytokines TNF-α, IL-1β, and IL-6 have been reported to be raised in rodents that exhibited a depression-like phenotype [66]. The effects of peripheral inflammation or immunological challenges do not remain in the periphery and may disrupt the brain blood barrier (BBB), allowing monocytes and cytokines to infiltrate through leaky regions of the BBB or through active transport systems [67]. Upon entering the brain, IL-1β is believed to be involved in compromising BDNF-mediated functions such as synaptic plasticity and neuronal survival [68]. In our study, EGCG significantly decreased the IL-1β serum concentration, indicating that it might elicit its antidepressant effect by reducing the inflammatory cytokine concentration. This finding is supported by the work of Chen et al. [69], which showed that EGCG reduced IL-1β expression at the mRNA level in the hippocampus of depressive mice.

Chronic stress causes downregulation of BDNF protein and mRNA in the hippocampus [70], which may induce depressive pathology [71, 72]. Classical antidepressants (e.g., selective serotonin reuptake inhibitors, SSRIs) increase the level of BDNF, which is highly correlated with symptom relief [73]. Moreover, direct infusion of BDNF into the hippocampus has shown antidepressant-like effects and decreased immobility in the FST [74]. In our study, RT–PCR quantification indicated a marked diminution of hippocampal BDNF mRNA in CUMS mice, which was significantly reversed after treatment with EGCG, suggesting that EGCG may exert neuroprotective and antidepressant effects, at least in part, through BDNF-related pathways, though the detailed mechanism requires future clarification. EGCG promotes neuronal development and synaptogenesis within the CNS, probably due to its ability to activate CREB [75], as BDNF is a target for the CREB transcription factor family [76].

So far, publications have not used TEM to analyze the in vivo effects of EGCG on CA3 neuronal alterations induced by CUMS. We showed that the subcellular pathology within the hippocampus of CUMS-exposed mice appeared mostly severe, with CA3 neurons featuring nuclear karyopyknosis (apoptosis), axonal demyelination and synaptic damage. These results are in line with previous studies [77–79], indicating that CA3 area is vulnerable to stress. In addition, in our research, we found that EGCG relieved the CA3 neuronal disorders in CUMS mice, corroborating earlier reports exploring the beneficial role of EGCG against neurodegenerative structural changes in the hippocampus [80, 81]. It has also been shown that in vitro administration of 0.5 µM EGCG may be effective in promoting axonal regeneration in cultured CNS cells and blocking the antineuritogenic activity of myelin-derived inhibitors [82]. Moreover, Menard et al. [83] provided in vitro clue that EGCG can inhibit neuronal cell death and promote synaptic development.

## Conclusion

**Fig. 6:**
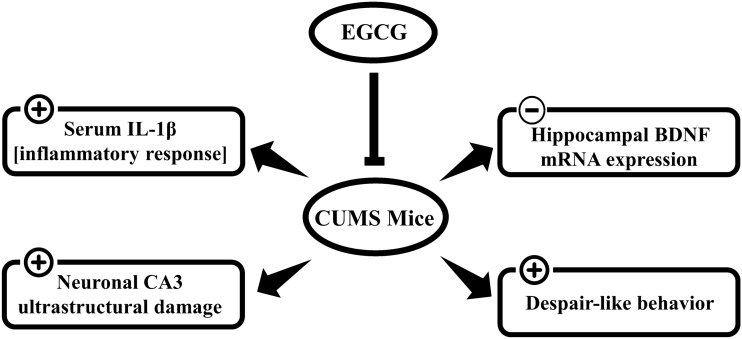
Schematic diagram showing the antidepressant actions of EGCG in stressed mice

Taken together, our results indicate that treatment with EGCG can decrease the immobility time in the FST (i.e., lower the levels of despair-like behavior). The present findings show that the antidepressant-like effect of EGCG is associated with an inhibitory action on systemic IL-1β, increased BDNF mRNA levels in the hippocampus and reduced neurologic injury caused by CUMS (Fig. 6). Some limitations of this study need to be addressed later. Here, we focused on BDNF mRNA, and it may be beneficial to investigate whether BDNF protein isoforms, i.e., pro- and mature-BDNF, are modified throughout the EGCG treatment and how early changes in BDNF isoform proportion might predict EGCG antidepressant-like actions. Our model may have applications for the development of herbal and safe therapy for MDD; however, to provide more robust evidence, additional research is needed to elaborate the effects of varied EGCG loads (i.e., dose and duration) on the neurobiological manifestations of depression.

## Data Availability

The datasets generated during and/or analysed during the current study are available from the corresponding author on reasonable request.
